# The Nature of Activatory and Tolerogenic Dendritic Cell-Derived Signal 2

**DOI:** 10.3389/fimmu.2014.00042

**Published:** 2014-02-06

**Authors:** Francesca Granucci, Manfred B. Lutz, Ivan Zanoni

**Affiliations:** ^1^Department of Biotechnology and Bioscience, University of Milano-Bicocca, Milan, Italy; ^2^Institute of Virology and Immunobiology, University of Wuerzburg, Wuerzburg, Germany

**Keywords:** dendritic cells, T cell priming, tolerance, costimulatory molecules, danger signals, regulatory T cells

Dendritic cells (DCs) were first described by Steinman and Cohn in 1973 (1) as cells provided of efficient antigen-presenting capacity. Steinmann received the Nobel Prize in 2011 for having revealed the pivotal role of DCs in linking innate and adaptive immunity and initiating antigen-specific immunity (2). The work conducted by Steinman was also instrumental in determining the role of DCs not only in activating adaptive immunity but also in controlling adaptive anti-self reactions by inducing and maintaining self-tolerance both at central and peripheral level (3).

Dendritic cells are highly heterogeneous. Two major classes of DCs have been described: classical or conventional and plasmacytoid DCs (pDCs). Classical DCs are cells of myeloid origin capable of efficiently capturing microbial antigens, and, after homing to lymph nodes and antigen processing, present these antigens to prime naive T cells. Diversely, pDCs are poorly phagocytic but recognize viruses very well. Upon encounter of microbial stimuli, they also differentiate into efficient antigen-presenting cells and express high levels of type I interferons (IFNs) (4). Based on tissue localization, transcription factor, and surface markers expression, classical DCs have been subdivided in a continuously growing number of subtypes (5, 6). Subsets expressing CD4/CD11b or CD8α/CD103 resides in secondary lymphoid organs; under healthy conditions lymph nodes contain additional subsets of partially matured steady state migratory DCs that transport self-antigens into lymph nodes for tolerance induction (7–10).

The DC capacity of inducing both immunity and tolerance may seem a contradictory aspect of DC biology, nevertheless the acquisition of these two different properties may depend on stimuli DC are exposed to or may be a specific feature of different DC subsets.

Dendritic cells sense the presence of exogenous microbial signals through germline-encoded pattern-recognition receptors (PRRs), which recognize molecular patterns expressed by various microorganisms or endogenous danger signals. The consequences of the activation of these receptors on DCs have been implicated in the acquisition of the immunogenic functions characterized by the increase of antigen presentation and costimulation as well as the release of proinflammatory cytokines. The attainment of the tolerogenic function by DCs seems instead to be more linked to the exposure to endogenous factors sensed in peripheral tissues under steady state conditions.

In the present Research topic, contributing articles describe a number of aspects determining the tolerogenic or immunogenic functions of DCs. In particular, the themes that will be discussed concern the role of DCs in controlling the threshold of activation of T cells; the role of the diverse costimulatory molecules, either secreted by DC subsets or expressed on the cell surface, in determining the DC immunogenic or tolerogenic functions; the role of endogenous or exogenous stimuli in influencing the DC functional state; some specific roles of DCs in preventing particular organ autoimmunity and, finally, possible therapeutic potentials of immunogenic or tolerogenic DCs.

Understanding the mechanisms that regulate the functional properties of DCs will allow exploiting these cells in new effective therapeutic strategies, including cancer immunotherapy and control of autoimmunity, to improve intervention outcomes. Understanding DC biology and their responses to activating stimuli will also help the identification of novel adjuvants to be used in new vaccine formulations.
